# miR-3913-3p promoted the progression of lung adenocarcinoma by regulating STX3 expression

**DOI:** 10.1186/s41065-026-00654-1

**Published:** 2026-03-04

**Authors:** Yu Wang, Lin Shen, Ruijie Cao, Hao Xu, Zhong Guo, Yuxing Chen, Dan Wang

**Affiliations:** 1 Institute of Upper Gastrointestinal Tumour Prevention and Treatment, Shanxi Medical University, Changzhi, 046000 China; 2 Department of Pathology, Changzhi People’s Hospital, The Affiliated Hospital of Changzhi Medical College, Changzhi, 046000 China; 3 Integrated Traditional Chinese and Western Medicine, Qingdao Central Hospital, University of Health and Rehabilitation Sciences (Qingdao Central Hospital), Qingdao, 266042 China; 4 Department of Respiratory Medicine, Shanghai Sixth People’s Hospital Jinshan Branch, Shanghai, 201599 China; 5 Department of Respiratory and Critical Care Medicine, Huanggang Central Hospital, No. 6, Qi’an Avenue, Huangzhou District, Huanggang, 438000 China

**Keywords:** miR-3913-3p, STX3, LUAD, Proliferation, Migration, Invasion

## Abstract

**Background:**

The prognostic relevance and operational pathways of miR-3913-3p during disease advancement are not entirely clarified. This research intends to thoroughly assess the clinical importance and mechanistic contributions of miR-3913-3p within lung adenocarcinoma (LUAD) contexts.

**Methods:**

Relationships linking miR-3913-3p abundance with five-year survival rates were examined through Kaplan-Meier methodology, while prognostic strength was determined by multivariate Cox regression modeling. Quantification of miR-3913-3p and STX3 in clinical specimens and cellular models was accomplished via RT-qPCR. Functional impacts on LUAD cells and regulatory interactions with target genes were validated through cell transfection, CCK-8 assays, Transwell migration/invasion assays, and dual-luciferase reporter assays.

**Results:**

miR-3913-3p expression was significantly elevated in LUAD tissues compared to matched non-tumor tissues. High miR-3913-3p expression was significantly correlated with poor tumor differentiation, advanced TNM stage, and lymph node metastasis (*p* < 0.05). Multivariate Cox analysis identified high miR-3913-3p expression as a predictor of poor prognosis (HR = 2.450, 95% CI: 1.014–5.920, *p* = 0.046). Functionally, miR-3913-3p mimic enhanced the proliferative, migratory, and invasive capacities of LUAD cells, whereas miR-3913-3p inhibitor suppressed these malignant behaviors. Notably, co-transfection with si-STX3 rescued the inhibitory effects induced by the miR-3913-3p inhibitor. Mechanistically, dual-luciferase reporter assays confirmed that miR-3913-3p directly binds to the 3’untranslated region (3’UTR) of STX3, leading to its functional suppression.

**Conclusion:**

This study demonstrates the existence of a regulatory miR-3913-3p/STX3 axis in LUAD. miR-3913-3p likely promotes the proliferation, migration, and invasion of LUAD cells by targeting STX3, implicating this axis in LUAD pathogenesis.

**Supplementary Information:**

The online version contains supplementary material available at 10.1186/s41065-026-00654-1.

## Background

Presently, pulmonary carcinoma represents a predominant neoplasm worldwide and constitutes a major source of cancer-associated fatalities, substantially compromising life quality [1]. Non-small cell lung cancer encompasses nearly 85% of pulmonary malignancy incidents [2], wherein lung adenocarcinoma (LUAD) constitutes the chief histological variant, representing above 50% of occurrences [3] and occurring more frequently in non-smokers. Treatment modalities for LUAD include surgical resection, radiotherapy, chemotherapy, immunotherapy, and their combinations [4]. Nevertheless, despite substantial advancements in therapeutic strategies in recent years, the overall 5-year survival rate for patients with LUAD remains below 20%, indicating a persistently poor prognosis [5]. Consequently, there is an urgent need to elucidate the mechanisms underlying LUAD malignant progression, particularly focusing on the regulatory networks that govern key cellular behaviors such as proliferation, migration, and invasion. Research in this direction is crucial for identifying novel prognostic biomarkers and therapeutic targets to improve clinical outcomes.

MicroRNAs, a class of short non-coding RNAs, regulate more than half of the genes in the human genome, where a single miRNA can influence a multitude of downstream transcripts [6]. Substantial research has illuminated the decisive regulatory functions of miRNAs during LUAD pathogenesis. Representative cases comprise miR-96-5p accelerating disease progression through ARHGAP6 downregulation [7]; miR-576-3p constraining cellular translocation and invasion via SGK1 repression [8]; miR-184 adjusting multiplicative, migratory, and invasive attributes by targeting C1QTNF6 [9]; miR-21-5p intensifying aggressive conduct through WWC2 inhibition, proposing innovative therapeutic strategies [10]; and miR-186-5p, which demonstrates upregulation in LUAD, stimulates neoplastic expansion and dissemination by suppressing PTEN [11]. Although existing literature indicates that miR-3913-3p is significantly upregulated in LUAD [12], this finding suggests that miR-3913-3p may potentially contribute to alterations in LUAD cell function, thereby influencing the biological functions of these cells and participating in the initiation and progression of the disease. However, the current understanding of the specific role and underlying mechanisms of miR-3913-3p in lung cancer remains incomplete.

MiRNAs typically exert their biological functions by binding to the complementary sequences on the3’ UTRs of their target mRNAs [13]. In lung cancer, STX3 functions as a protective gene, and its downregulation is associated with poorer prognosis, while its high expression correlates with improved patient survival [14]. This study aims to systematically explore the expression patterns, biological functions, and underlying molecular mechanisms of miR-3913-3p in LUAD. Our findings are expected to enhance the understanding of the molecular significance of miR-3913-3p in LUAD progression and provide new potential targets for clinical diagnosis and treatment.

## Methods

### Study subjects

A cohort of 120 patients newly diagnosed with LUAD who underwent surgery at Shanghai Sixth People’s Hospital Jinshan Branch was enrolled in this study. All patients were pathologically confirmed prior to enrollment and had not received any antitumor therapy before surgery. Neoplastic and adjoining normal tissues were acquired during operative interventions from all enrolled individuals. The experimental protocol obtained authorization from the Institutional Review Board of Shanghai Sixth People’s Hospital Jinshan Branch, with written informed consent secured from each participant under alert circumstances. Postoperative survival status was actively monitored via scheduled telephone follow-ups for up to 5 years, death as the follow-up endpoint. Tumor staging adhered to the UICC TNM classification [15], categorizing patients into stages I-Ⅲ. Tumor differentiation degree was assessed based on postoperative pathological sections: tumors with morphology and structure close to normal cells were considered well-differentiated, while those markedly different from normal cells were considered poorly differentiated.

Inclusion criteria were: (1) adult patients aged 18–75 years; (2) complete medical records and follow-up data; (3) no history of relevant drug or surgical treatment. Exclusion criteria included: patients with mixed adenocarcinoma, metastatic malignant tumors, coagulation disorders, cardiopulmonary insufficiency, and mental illnesses.

### Cell transfection

Cellular transfection procedures employed the human bronchial epithelial line BEAS-2B alongside LUAD derivatives (H1299, A549, H1975, PC-9), sourced from the Shanghai Institute of Biological Sciences, CAS. Cultivation occurred in Dulbecco’s Modified Eagle Medium containing 10% fetal bovine serum at 37 °C within 5% CO_2_ humidified conditions. miR-3913-3p mimic, inhibitor, small-interfering RNA (si-STX3), and respective negative controls were procured from GeneChem. Transfection into A549 and PC-9 cells utilized Lipofectamine 3000, with cellular collection after 48 h for downstream analyses.

### Quantitative real-time PCR

Total RNA was extracted from serum and cells with Trizol reagent (Invitrogen, USA). After assessing the purity and concentration of the RNA, high-quality RNA was used for reverse transcription to generate complementary DNA (cDNA). PCR amplification was then performed using the corresponding primers on a Roche PCR system (Switzerland). The PCR conditions were as follows: initial denaturation at 95 °C for 10 min, followed by 40 cycles of 95 °C for 15 s and 60 °C for 1 min. Normalization relied on U6 and GAPDH, with relative expression computed via the 2^−ΔΔCT^ method. The primer sequences are shown in Supplementary Table 1.

### Cell Counting Kit-8 (CCK-8)

Proliferation measurements utilized the Cell Counting Kit-8. Transfected A549 and PC-9 cells were distributed into 96-well plates at 3 × 10³ cells/well and maintained at 37 °C. Following 24, 48, and 72-hour intervals, 10 µl CCK-8 solution was introduced per well, with subsequent 2-hour incubation. Optical density readings were taken at 450 nm.

### Transwell

Cell migration and invasion assays were performed using a Boyden chamber system (Neuro Probe, Gaithersburg, MD, USA). A polycarbonate membrane (8 μm pore size) was placed between the upper and lower chambers. For the invasion assay, the upper surface of the membrane was pre-coated with 1 µg/µL Matrigel matrix (BD Biosciences, Bedford, MA, USA), whereas an uncoated membrane was used for the migration assay. Cells were trypsinized, centrifuged, and resuspended in DMEM supplemented with 10% fetal bovine serum, followed by cell counting. Subsequently, cells were seeded into the upper chamber at a density of 4 × 10⁵ cells per well. An identical medium was added to the lower chamber as a chemoattractant. After 24 h of incubation at 37 °C under 5% CO₂, the chambers were fixed with methanol and stained with Giemsa solution (Sigma-Aldrich). Non-migrated/non-invaded cells on the upper surface of the membrane were carefully removed, and cells that had migrated or invaded to the lower surface of the membrane were counted for statistical analysis.

### Dual-luciferase reporter assay

A549 and PC-9 cells were seeded in 24-well plates 24 h prior to transfection. The wild-type vector contained the full-length 3′-UTR sequence of the target gene, while the mutant vector was generated by introducing site-directed mutations into the putative miR-3913-3p binding site, followed by in vitro recombination. The reporter vectors were co-transfected with miR-3913-3p mimic, miR-3913-3p inhibitor, or negative controls using Lipofectamine 3000 (Invitrogen, USA) transfection reagent. Luciferase activity was measured 48 h post-transfection using a dual-luciferase reporter assay system (Yeasen Biotechnology, China).

### Statistical analysis

Statistical analysis was performed using SPSS and GraphPad Prism 9. All experiments were independently repeated three times. Quantitative results are expressed as mean ± standard deviation. Comparisons between two groups were conducted using Student’s t-test, while comparisons among multiple groups were performed using one-way ANOVA followed by Tukey’s post hoc test to assess intergroup differences. Overall survival was analyzed using the Kaplan‑Meier method. Univariate and multivariate Cox proportional hazards regression analyses were employed to identify independent risk factors associated with the prognosis of lung adenocarcinoma. The correlation between miR‑3913‑3p and STX3 expression was assessed using Pearson correlation analysis. A threshold of *p* < 0.05 was considered statistically significant.

## Results

### Association between miR-3913-3p expression and clinicopathological characteristics in LUAD patients

**Fig. 1:**
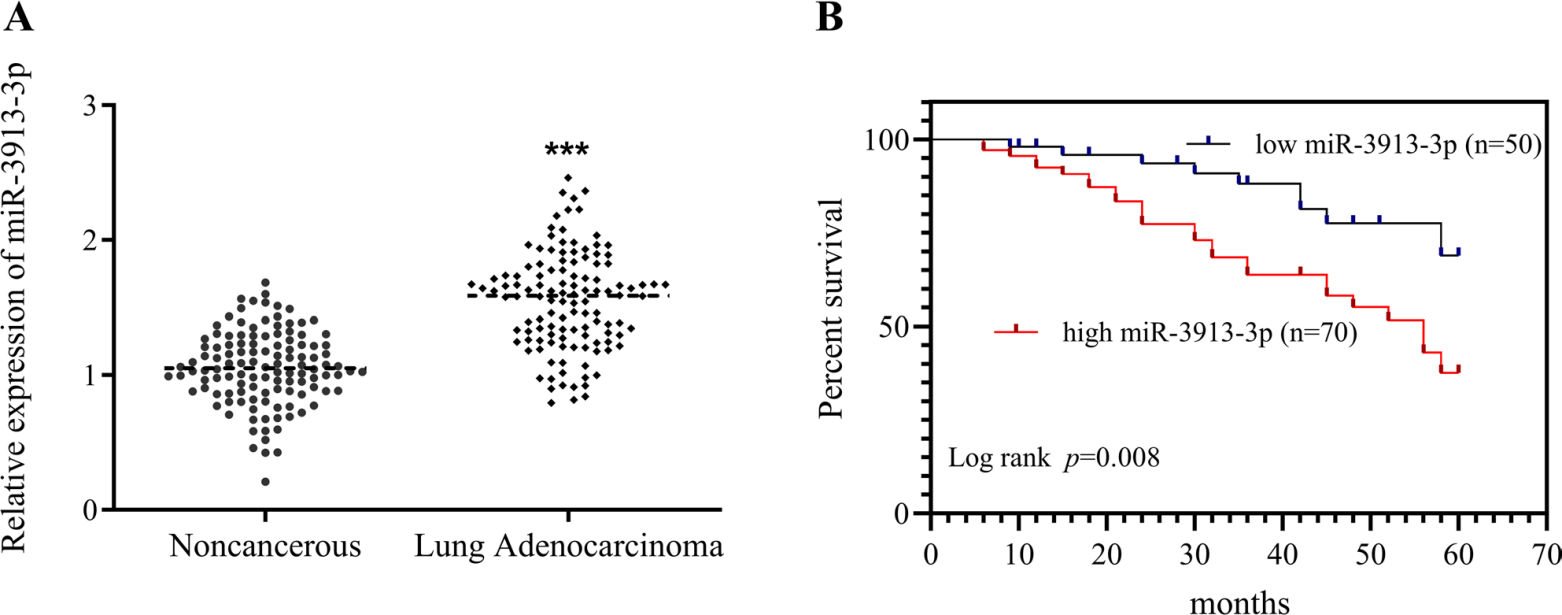
The clinical significance of miR-3913-3p in LUAD. **A** Relative expression level of miR-3913-3p in LUAD tissues compared to adjacent noncancerous tissues as determined by RT-qPCR. **B** Kaplan-Meier overall survival curves of LUAD patients with high versus low miR-3913-3p expression (*p* = 0.008). ***p < 0.001

RT-qPCR analyses demonstrated pronounced miR-3913-3p elevation in LUAD tissues compared to normal adjacents (Fig. 1A). Based on the median expression level of miR-3913-3p in LUAD tissues, the collected tissue samples were dichotomized into a low-expression group (*n* = 50) and a high-expression group (*n* = 70). Associations between miR-3913-3p expression and clinicopathological characteristics of LUAD patients were examined using the chi-square test. Evaluation of 120 LUAD cases indicated miR-3913-3p levels lacked significant associations with sex (*p* = 0.779), age (*p* = 0.267), smoking status (*p* = 0.068), or lesion dimensions (*p* = 0.949). Conversely, the high-expression subset exhibited substantially greater frequencies of nodal metastasis (*p* = 0.028), advanced TNM stage (*p* = 0.034), and poor differentiation (*p* = 0.006) relative to the low-expression cohort (Table 1), suggesting a close association between high miR-3913-3p expression and malignant progression of LUAD.

**Table 1:** Association between miR-3913-3p level and pathologic characteristics in patients with lung adenocarcinoma

Parameters		miR-3913-3p expression			*p*-value
		Total	High	Low	
		120	70	50	
Gender	Male	69	41	28	0.779
	Female	51	29	22	
Age (years)	< 60	60	38	22	0.267
	≥ 60	60	32	28	
Smoking history	No	65	33	32	0.068
	Yes	55	37	18	
Tumor size (mm)	≤ 30	46	27	19	0.949
	> 30	74	43	31	
Differentiation	Well-moderate	64	30	34	0.006*
	Poor	56	40	16	
Neoplasm stage	T1/T2	81	47	34	0.921
	T3/T4	39	23	16	
TNM stage	Ⅰ-Ⅱ	73	37	36	0.034*
	Ⅲ	47	33	14	
Lymph node metastasis	N0	75	38	37	0.028*
	N1/N2/N3	45	32	13	

### Prognostic value of miR-3913-3p for LUAD

During the 5-year follow-up period, Kaplan-Meier curves were generated to assess the association between miR-3913-3p expression and prognosis in patients with LUAD. High expressers experienced considerably shorter overall survival (Fig. 1B). Univariate Cox regression analysis revealed that elevated miR-3913-3p expression (HR = 3.094, 95% CI: 1.337–7.159, *p* = 0.008), tumor differentiation grade (HR = 2.150, 95% CI: 1.057–4.372, *p* = 0.035), and lymph node metastasis (HR = 2.049, 95% CI: 1.022–4.110, *p* = 0.043) were significantly associated with poor patient prognosis. Furthermore, multivariate Cox regression analysis confirmed that high miR-3913-3p expression (HR = 2.450, 95% CI: 1.014–5.920, *p* = 0.046) served as an independent risk factor for unfavorable prognosis in LUAD patients (Table 2).

**Table 2:** Univariate and multivariate analyses of clinical characteristics associated with overall survival of lung adenocarcinoma patients

Variable	Univariate analysis			Multivariate analysis		
	HR	95% CI	*P*-value	HR	95% CI	*P*-value
miR-3913-3p	3.094	1.337–7.159	0.008^*^	2.450	1.014–5.920	0.046^*^
Gender	1.224	0.604–2.480	0.575			
Age	1.141	0.558–2.329	0.718			
Smoking history	1.661	0.801–3.446	0.173			
Tumor size	1.115	0.536–2.322	0.771			
Differentiation	2.150	1.057–4.372	0.035*	1.729	0.833–3.589	0.141
Neoplasm stage	1.197	0.565–2.534	0.631			
TNM stage	1.709	0.853–3.424	0.131			
LNM	2.049	1.022–4.110	0.043*	1.452	0.702–3.005	0.315

*TNM* Tumor, Node, Metastasis, *LNM* Lymph node metastasis,*P<0.05

### miR-3913-3p stimulates LUAD cell proliferation, migration, and invasion

**Fig. 2:**
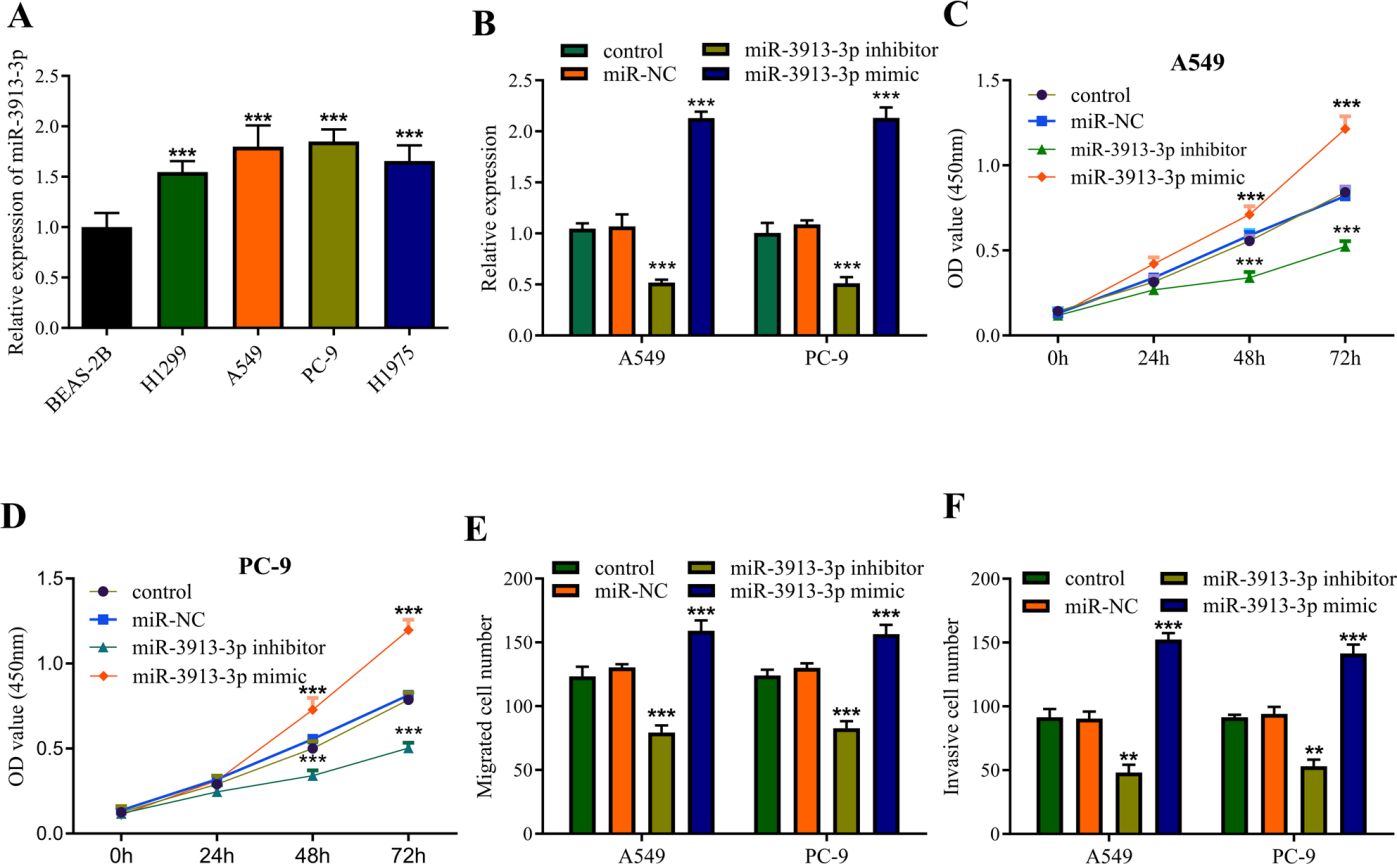
Effects of miR-3913-3p on the proliferation, migration, and invasion abilities of LUAD cells. **A** Relative expression levels of miR-3913-3p in BEAS-2B and a panel of LUAD cell lines (H1299, A549, PC-9, H1975) as determined by RT-qPCR. **B** Validation of miR-3913-3p expression in A549 and PC-9 cells after transfection with miR-3913-3p mimic or inhibitor. **C** Proliferation capacity of A549 cells following modulation of miR-3913-3p expression, as assessed by the CCK-8 assay. **D** Proliferation capacity of PC-9 cells following modulation of miR-3913-3p expression, as assessed by the CCK-8 assay. **E** Transwell assays assessing the migratory abilities of A549 and PC-9 cells following modulation of miR-3913-3p expression. **F** Transwell assays assessing the invasive abilities of A549 and PC-9 cells following modulation of miR-3913-3p expression. **p < 0.01, ***p < 0.001

To verify the clinical relevance, the expression pattern of miR-3913-3p was examined in LUAD cell lines (H1975, PC‑9, A549, H1299) and the normal bronchial epithelial cell line BEAS‑2B. RT‑qPCR results showed that miR-3913-3p was consistently upregulated in all cancer cell lines compared to BEAS‑2B cells (Fig. 2A), with the most pronounced differential expression observed in PC‑9 and A549 cells. Therefore, these two cell lines were selected for subsequent investigations. To further verify whether miR-3913-3p promotes LUAD cell progression, transfection of PC-9 and A549 cells with miR-3913-3p mimic or inhibitor constructs confirmed anticipated expression alterations through RT-qPCR verification, indicating successful transfection (Fig. 2B). Subsequently, CCK-8 assays demonstrated that transfection with miR-3913-3p mimic significantly enhanced the proliferative capacity of LUAD cells, while knockdown of miR-3913-3p led to significantly reduced OD values in PC-9 and A549 cells at 48 and 72 h post-seeding, indicating suppressed proliferation (Fig. 2C, D). Transwell migration and invasion protocols indicated that miR-3913-3p overexpression potentiated cellular motility and infiltration, while its suppression impaired these capabilities (Fig. 2E, F). These experiments robustly confirm that miR-3913-3p stimulates malignant processes in LUAD cells.

### Relationship between miR-3913-3p and STX3 in LUAD

**Fig. 3:**
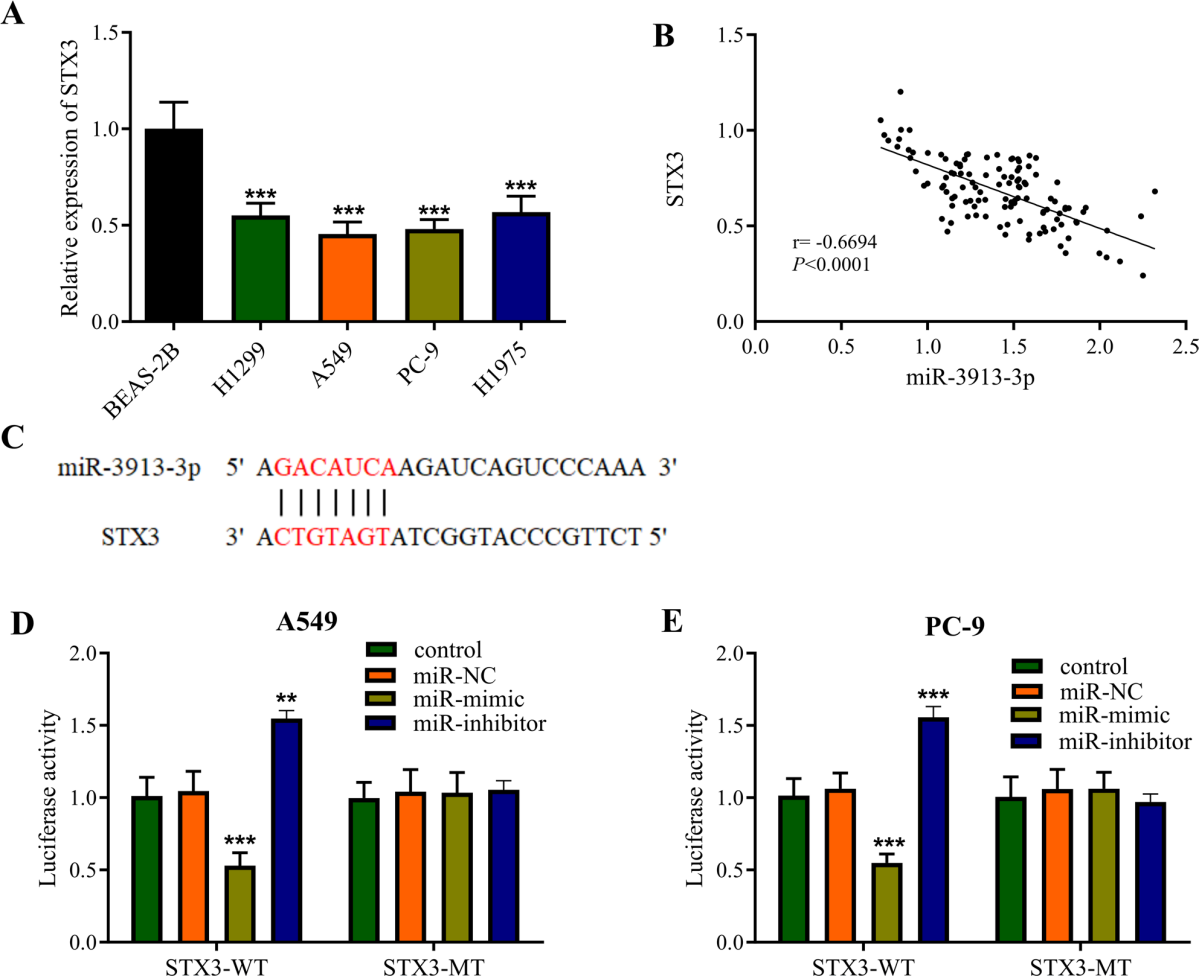
miR-3913-3p directly targets and inhibits STX3 expression in LUAD. **A** Relative expression level of STX3 in LUAD tissues compared to adjacent noncancerous tissues as determined by RT-qPCR. **B** Scatter plot showing a significant negative correlation (r = -0.669, *p* < 0.0001) between miR-3913-3p and STX3 expression levels in LUAD tissues. **C** Schematic diagram of the predicted binding site for miR-3913-3p within the 3’ untranslated region (3’UTR) of STX3. **D** Dual-luciferase reporter assay in A549 cells. **E** Dual-luciferase reporter assay in PC-9 cells. **p < 0.01, ***p < 0.001

STX3 expression proved significantly diminished in LUAD cell lines compared to HBE controls (Fig. 3A). Inverse correlation emerged between miR-3913-3p and STX3 levels in patient-derived tumors (*r* = -0.669, Fig. 3B). Bioinformatics interrogation using StarBase nominated STX3 as a prospective miR-3913-3p target, consistent with its tumor suppressor characteristics in LUAD. Predicted binding motifs appear in Fig. 3C. Dual-luciferase assays documented marked activity reduction in LUAD (A549 and PC-9) cells containing wild-type STX3 3’UTR upon miR-3913-3p mimic introduction, whereas activity was significantly increased following introduction of the miR-3913-3p inhibitor. In contrast, no significant effect was observed on the mutant (STX3-MUT) reporter (Fig. 3D, E). These results indicate that miR-3913-3p can directly interact with STX3.

### miR-3913-3p promotes LUAD cell proliferation, migration, and invasion by suppressing STX3

**Fig. 4:**
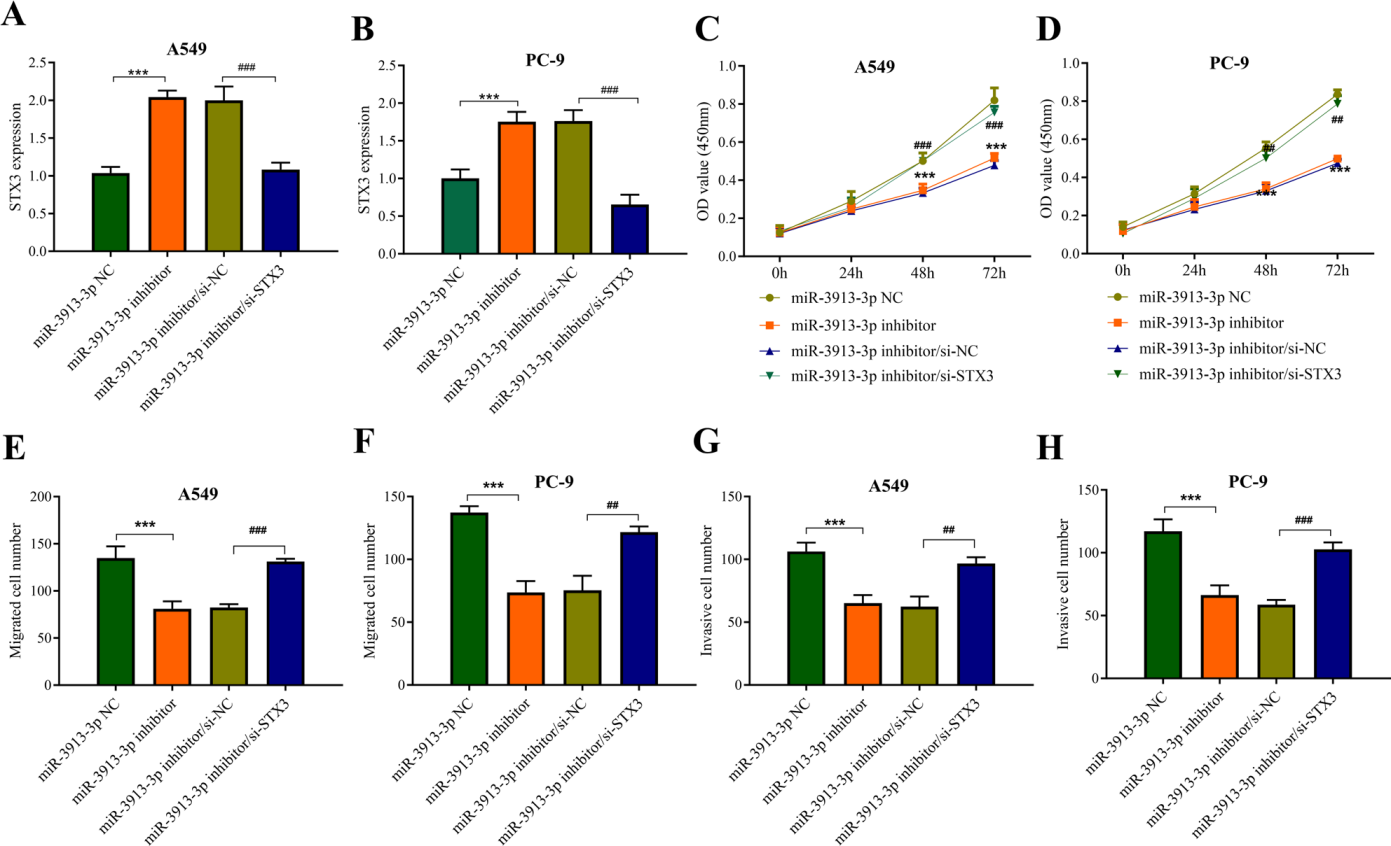
STX3 knockdown reverses the tumor-suppressive effects of miR-3913-3p inhibition in LUAD cells. **A** Relative mRNA expression level of STX3 in A549 cells from different treatment groups. **B** Relative mRNA expression level of STX3 in PC-9 cells from different treatment groups. **C** Cell proliferation assessed by CCK-8 assay in A549 cells transfected with miR-3913-3p inhibitor alone or co-transfected with si-STX3. **D **Cell proliferation assessed by CCK-8 assay in PC-9 cells transfected with miR-3913-3p inhibitor alone or co-transfected with si-STX3. **E** Transwell migration assays in A549 cells showing that silencing STX3 partially rescues the impaired migration and invasion caused by miR-3913-3p inhibition. **F** Transwell migration assays in PC-9 cells showing that silencing STX3 partially rescues the impaired migration and invasion caused by miR-3913-3p inhibition. **G** Invasion assays in A549 cells showing that silencing STX3 partially rescues the impaired migration and invasion caused by miR-3913-3p inhibition. **H** Invasion assays in PC-9 cells showing that silencing STX3 partially rescues the impaired migration and invasion caused by miR-3913-3p inhibition. **p < 0.01, ***p < 0.001, ^##^*p* < 0.01, ^###^*p* < 0.001

Rescue experiments were conducted to verify whether the oncogenic effect of miR-3913-3p is mediated through the suppression of STX3. In both A549 and PC-9 cell lines, co-transfection with si-STX3 effectively countered the upregulation of STX3 mRNA induced by the miR-3913-3p inhibitor, returning expression to baseline levels (Fig. 4A, B). Functionally, knockdown of miR-3913-3p significantly suppressed the proliferation of both A549 and PC-9 cells, and this effect was reversed by concurrent STX3 knockdown (Fig. 4C, D). Similarly, knockdown of miR-3913-3p markedly reduced the migratory and invasive capacities of the cells, and this suppression was effectively rescued by co‑knockdown of STX3 (Fig. 4E-H). These results demonstrate that miR-3913-3p promotes proliferation, migration, and invasion in LUAD by directly repressing STX3.

## Discussion

As pivotal gene expression modulators, miRNAs significantly influence tumor initiation, progression, and dissemination [16, 17]. The miR-3913 family includes two major mature isoforms: miR-3913-3p and miR-3913-5p. Emerging evidence reveals that miR-3913-5p exhibits context-dependent roles in human cancers. In cholangiocarcinoma (CCA), it functions as a potential oncogene, where its upregulation promotes tumor cell proliferation and migration, suggesting its utility as a diagnostic and prognostic biomarker [18]. Conversely, in colorectal cancer (CRC), miR-3913-5p acts as a tumor suppressor through direct targeting of CREB5, forming a coherent regulatory circuit with ATF2 that critically modulates CRC progression [19]. Additionally, bioinformatics analyses in hepatocellular carcinoma have identified miR-3913-5p among key prognostic microRNAs, with its predicted targets enriched in crucial pathways including apoptosis, inflammation, and cell cycle regulation [20]. However, the functional roles and regulatory mechanisms of miR-3913-3p remain poorly characterized. Our inquiry concentrates on miR-3913-3p’s expression and operational dynamics in LUAD. Assessment of 120 patient samples uncovered substantial miR-3913-3p upregulation in malignant tissues, with high expression correlating with adverse pathological indicators including nodal metastasis, advanced staging, and poor differentiation. These clinical observations propose miR-3913-3p as a significant facilitator of LUAD malignancy. The upregulation of miR-3913-3p and its association with advanced stage and poor prognosis in lung adenocarcinoma follow a pattern reminiscent of the role reported for B3GNT3 in lung cancer, where its overexpression is also correlated with adverse clinicopathological features and promotes tumor progression [21]. Subsequent survival evaluation showed pronounced overall survival reduction among high expressers, with multivariate Cox verification as a prognostic determinant, supporting its utility as a potential prognostic indicator.

Functional studies authenticated the oncogenic capacity of miR-3913-3p across diverse LUAD lines. Experimental outcomes demonstrated that miR-3913-3p overexpression considerably accelerated proliferative, migratory, and invasive functions, while its knockdown effectively mitigated these malignant characteristics. This consistency with clinical observations reinforces miR-3913-3p’s central role in LUAD pathogenesis. Contemporary reports describe dysregulated miR-3913-3p activity in other malignancies; for example, miR-3913-5p shows marked upregulation in CCA, where elevated expression correlates with inferior prognosis and serves as an independent prognostic determinant by potentially targeting SIGLEC10 and RNF24 to stimulate proliferation and migration [22]. This analogous oncogenic behavior aligns with our findings, suggesting conserved pathological mechanisms across cancer types. These studies provide valuable references for our understanding of the role of miR-3913-3p in LUAD.

Mechanistic exploration identified STX3 as a direct miR-3913-3p target in LUAD. The Syntaxin protein family encompasses 16 recognized members, spanning STX1A-1B through STX8, STX10 through STX12, and STX16 through STX19 [23]. STX3 operates as an apical receptor coordinating vesicular membrane fusion [24]. Mammary carcinoma, STX3 stimulates neoplastic growth and may indicate unfavorable prognosis [25, 26]. Correspondingly, esophageal squamous cell carcinoma exhibits heightened STX3 mRNA association with nodal engagement, advanced pathology, and diminished survival, proposing its biomarker utility [27]. Our investigation validated direct miR-3913-3p binding to the STX3 3’UTR through computational prediction and dual-luciferase assays. LUAD cellular models displayed significantly reduced STX3 levels versus normal pulmonary epithelia, exhibiting negative correlation with miR-3913-3p expression. These results collectively suggest that STX3 exhibits a context-dependent regulatory pattern. The functional divergence of this molecule across different cancer types may stem from tissue-specific expression profiles, distinct cellular microenvironments, and differential availability of interacting partners such as miRNAs or RNA-binding proteins [28]. Such functional complexity underscores the necessity to investigate STX3 biology within specific pathological contexts, as its roles are likely intimately linked to the unique molecular landscape characterizing each cancer type. More importantly, rescue experiment results indicated that knocking down STX3 could partially reverse the anti-tumor effects caused by miR-3913-3p inhibition, which provides direct evidence for the regulatory mechanism whereby miR-3913-3p promotes LUAD progression by targeting STX3.

This study has certain limitations. As shown by Zhao et al., targeting miR-651-5p in a humanized mouse model of EGFR-mutant lung adenocarcinoma significantly enhanced CD8⁺ T cell infiltration and improved the anti-tumor efficacy of PD-1 inhibitors [29]. This finding underscores the importance of in vivo experiments for comprehensively evaluating therapeutic strategies and for elucidating tumor-immune microenvironment interactions. Due to constraints in the research timeline and available experimental conditions, this study did not include related animal in vivo experiments. Therefore, subsequent research will establish corresponding in vivo models to further validate the biological functions and therapeutic potential of miR-3913-3p, thereby providing a more substantial foundation for its translational application.

In summary, this study delineates the expression profile, clinical relevance, and functional role of miR‑3913‑3p in LUAD, and demonstrates that it promotes malignancy by directly targeting STX3. These findings suggest miR‑3913‑3p as a potential prognostic biomarker and provide a rationale for therapeutic strategies targeting the miR‑3913‑3p/STX3 axis.

## Supplementary Information

Supplementary Material 1.

## Data Availability

Data available on request from the authors.
