# Supplementary material for: miR-3913-3p promoted the progression of lung adenocarcinoma by regulating STX3 expression
Source: Hereditas. 2026 Mar 4;163:48. doi: 10.1186/s41065-026-00654-1 (PMC13067435; doi:10.1186/s41065-026-00654-1)
Supplement: Supplementary file 1 — Supplementary Material 1. [file 41065_2026_654_MOESM1_ESM.docx]

Supplementary Table1 The primer sequences used in this study

| Name | Sequences (5'-3') |  |
| --- | --- | --- |
| miR-3913-3p | Forward | GACATCAAGATCAGTCCCA |
|  | Reverse | TTTGGGACTGATCTTGATGT |
| STX3 | Forward | ACACGGCTTTTATGGACGAG |
|  | Reverse | CGTTGTTGGCCCTTTTCTTA |
| U6 | Forward | CGTTTTACTTCCTCATACAGCAC |
|  | Reverse | GCACCAAGAGACCTGTGACA |
| GAPDH | Forward | GAAGGTGAAGGTCGGAGTC |
|  | Reverse | GAAGATGGTGATGGGATTTC |
